# Properties and Mechanism of Hydroxyapatite Coating Prepared by Electrodeposition on a Braid for Biodegradable Bone Scaffolds

**DOI:** 10.3390/nano9050679

**Published:** 2019-05-02

**Authors:** Ting-Ting Li, Lei Ling, Mei-Chen Lin, Qian Jiang, Qi Lin, Jia-Horng Lin, Ching-Wen Lou

**Affiliations:** 1 Innovation Platform of Intelligent and Energy-Saving Textiles, School of Textile Science and Engineering, Tianjin Polytechnic University, Tianjin 300387, China; tingtingli@tjpu.edu.cn (T.-T.L.); 1731015027@stu.tjpu.edu.cn (L.L.); jiangqian@tjpu.edu.cn (Q.J.); 2 Tianjin and Ministry of Education Key Laboratory for Advanced Textile Composite Materials, Tianjin Polytechnic University, Tianjin 300387, China; 3 Fujian Key Laboratory of Novel Functional Fibers and Materials, Minjiang University, Fuzhou 350108, China; 4 School of Chinese Medicine, China Medical University, Taichung 40402, Taiwan; ritalin2870@mail.fcu.edu.tw; 5 Laboratory of Fiber Application and Manufacturing, Department of Fiber and Composite Materials, Feng Chia University, Taichung 40724, Taiwan; 6 Fujian Engineering Research Center of New Chinese Lacquer Material, Minjiang University, Fuzhou 350108, China; linqi@mju.edu.cn; 7 Ocean College, Minjiang University, Fuzhou 350108, China; 8 College of Textile and Clothing, Qingdao University, Qingdao 266071, China; 9 Department of Fashion Design, Asia University, Taichung 41354, Taiwan; 10 Department of Bioinformatics and Medical Engineering, Asia University, Taichung 41354, Taiwan; 11 Department of Medical Research, China Medical University Hospital, China Medical University, Taichung 40402, Taiwan

**Keywords:** hydroxyapatite, braid, electrodeposition, formation mechanism, roughness

## Abstract

Hydroxyapatite (HA) coating is successfully prepared by electrodeposition on the surface of polyvinyl alcohol (PVA)/polylactic acid (PLA) braid which serves as a potential biodegradable bone scaffold. The surface morphology, element composition, crystallinity and chemical bonds of HA coatings at various deposition times (60, 75, 90, 105 and 120 min) are characterized by scanning electron microscopy (SEM), energy dispersive X-ray analysis (EDAX), X-ray diffraction (XRD) and Fourier transform infrared spectroscopy (FTIR), respectively. Average Surface roughness (Ra) of HA coating is observed by confocal microscopy. The results reveal that the typical characteristic peaks of the FTIR spectrum confirm that HA coating is successfully prepared on the rugged surface of the PVA/PLA braid. The XRD results indicate that the crystallinity of HA can be improved by increasing deposition time. In the 90 min-deposition, hydroxyapatite has a dense and uniform coating morphology, Ca/P ratio of 1.7, roughness of 0.725 μm, which shows the best electrodeposition performance. The formation mechanism of granular and plate-like hydroxyapatite crystals is explained by the structural characteristics of a hydroxyapatite unit cell. This study provides a foundation for a bone scaffold braided by biodegradable fibers.

## 1. Introduction

Due to excellent mechanical property, biocompatibility, and biological inertia against the body fluid, bio-ceramics have been commonly used in medical implant materials [1,2,3]. Hydroxyapatite (Ca_10_(PO_4_)_6_(OH)_2_, HA) bio-ceramic which has good bioactivity and osteo-inductivity is a major component of human bones and teeth [4,5,6]. Therefore, hydroxyapatite coatings are usually prepared on the surface of clinical medical implants to facilitate the growth of newborn bones in organism [7,8]. Nowadays, the main bone scaffold materials that are widely evaluated are non-degradable metal materials such as titanium and its alloys, 316 L stainless steel, etc. [9,10]. Contrary to metallic bone scaffolds, the bone scaffolds composed of biodegradable fiber bundle braids enable the osteocyte proliferates with the braids being decomposed. When the impaired bones recover completely, the temporary braided scaffold is also totally decomposed, which attains spontaneous rehabilitation of the impaired bones.

In our previous study, we prepared three-layer polyvinyl alcohol (PVA) braid-structure bone scaffolds and confirmed that the porosity and water stability of the braid could be improved by crosslinking the braid with glutaraldehyde [11]. Further, we also fabricated a five-layer core-shell-structure PVA fiber bundles braided bone scaffold on which the hydroxyapatite crystal was directly coated by freeze-drying method and successfully help the recovery of damaged tibias of rats [12]. However, the freeze-drying process requires complex equipment and long process cycle, and the hydroxyapatite crystal is expensive and difficult to commercial application.

The RF-magnetron sputtering [13], the biomimetic method [14], sol-gel process [15], electrophoretic deposition [16] are all feasible preparation methods for hydroxyapatite (HA) coating, but some of these techniques are high cost, form uneven coating morphology and could not control the process precisely. For example, HA coating prepared by RF-magnetron sputtering is compact and uniform, but the preparation of target is complex and only one side can be deposited [17]. Unlike the aforementioned methods, electrochemical deposition is not limited to the substrate shape, and has more advantages of precise control of coating morphology, thickness, and chemical composition by changing ionic concentration of electrolyte, electrolyte composition, deposition current and deposition time [18,19,20].

Polyvinyl alcohol (PVA) and polylactic acid (PLA) fibers are two stand-out representative biodegradable fibers which are widely used in the preparation of biodegradable bone scaffolds [21,22,23]. In this study, we evaluate the performance of HA coating prepared by electrodeposition on PVA/PLA braid. HA coatings with different morphologies are prepared at different deposition time. The morphology of the coating is observed by SEM, and the elemental composition of the coating is analyzed by EDAX. Average surface roughness of HA coating is observed by confocal microscopy. The functional groups and chemical bonds of HA coating are characterized by FTIR, and XRD is used to analyze the phase composition and crystallinity of the coating. Finally, a time-dependent model for the HA crystal growth during electrodeposition is established from the viewpoint of the cell structure of an HA crystal. We hope this study can serve as a foundation of braid-structure scaffolds made of degradable fibers for future studies.

## 2. Materials and Methods

### 2.1. The Preparation of PVA/PLA Braids

**Figure 1:**
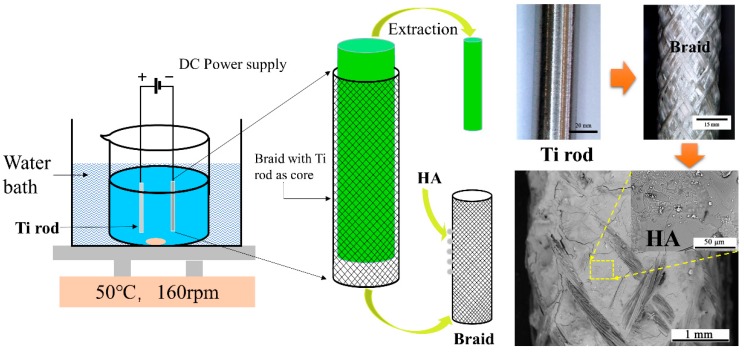
Images of PVA/PLA-HA composite braid prepared by electrodeposition.

PLA filaments (50 D/6f) and PVA filaments (38D/6f) with a blending ratio of 3:3 (mass ratio of 1:1) are combined and twisted into PVA/PLA composite yarns with 264 D/36 f fineness and 30 twists/10 cm. PVA/PLA yarns are provided by Beijing Guanghui Textile Co., Ltd, Beijing, China. Next, the composite yarns are heated for twist setting at 75 °C for 30 min, after which they are cooled to the room temperature. PVA/PLA composite yarns are braided into 4-mm-diamter five-layer braids using a 3-mm-diamteter titanium (Ti) rod as the supportive rod on a braiding machine (HSB-1, Dongguan Chenghong Electrical Material Co., Ltd., Dongguan, China), as shown in the right half of Figure 1. Then the composite braids are cleaned with ethanol, acetone, and water separately for 1 h. The composite braids are then dried at 65 °C for 3 h.

### 2.2. The Electrodeposition of HA Coatings

HA coating is prepared by traditional two-electrode system, as revealed in Figure 1. The anode is a 3-mm-diameter Ti rod and the cathode is the five-layer PVA/PLA braid. The electrolyte solution is composed of 1 mL/L 30 wt.% H_2_O_2_, 0.0048 M KH_2_PO_4_ and 0.008 M CaCl_2_ with a Ca/P ratio of 1.67. All reagents are analytical pure and provided by Tianjin Fengchuan Chemical Reagent Technology Co., Ltd., Tianjin, China. The DC power supply (IT6942A, ITECH, Nanjing, China) has a specified current of 0.01A. The distance between anode and cathode is 20 mm. The water bath temperature is 50 ℃ and the pH value of electrolyte is 4.3. The magnetic stirrer has a rotating speed of 160 rpm. The deposition time is 60, 75, 90, 105, and 120 min respectively.

### 2.3. The Characterization of HA Coatings

A field emission scanning electron microscope (FE-SEM, Gemini SEM500, Heidenheimer, Germany) equipped with an energy dispersive X-ray analyzer (EDAX, Octane Super, Mahwah, NJ, USA) is used to investigate the surface morphology and elemental composition of HA coatings on PVA/PLA braids. Before SEM observation, a layer of platinum is sprayed on the sample surface by an ion sputtering instrument (Blatc SCD005, BAL-TEC, Los Angeles, CA, USA). The chemical bonds of HA coating are observed by Fourier transform infrared spectroscope (FTIR, Nicolet iS50, Thermo Fisher Scientific, Waltham, MA, USA) at a resolution of 0.5 cm^−1^ over the wavenumber range of 400~4000 cm^−1^. True color confocal microscopy (Zeiss CSM700, Heidenheimer, Germany) is used to obtain the average surface roughness and 3D profilometry of the HA coating under a 20 times magnification. The measured area is 60 μm × 60 μm. Calcium ion concentration and total phosphorus content in the deposition process were measured by inductively coupled plasma atomic emission spectrometry (ICP-AES, PQ9000 Elite, Jena, Germany). Total phosphorus content includes hydrogen phosphate ion, dihydrogen phosphate ion and phosphate ion.

The phase composition of HA coatings is performed by X-ray diffraction (XRD, D8 Discover, Bruker, Karlsruhe, Germany) with a CuKα radiation (λ = 1.5405 Å) at 2 θ being 20–45° with a step size being 0.1°. Cell parameters and degree of crystallinity of HA crystals obtained by electrodeposition are estimated via Jade 6.0 software (MDI, Livermore, CA, USA). Meanwhile, the grain size of crystals obtained after a 90 min deposition is computed using the Debye–Scherrer equation according to Ref. [24].

## 3. Results and Discussion

### 3.1. The Surface Morphology and Element Composition of HA Coatings

**Figure 2:**
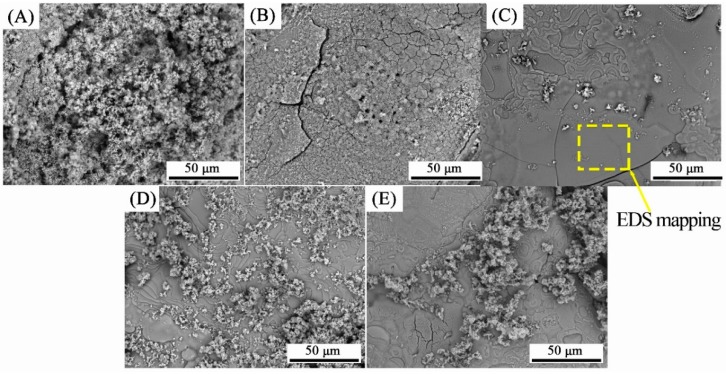
Surface morphology of HA coating on PVA/PLA braids with deposition time of (**A**) 60, (**B**) 75, (**C**) 90, (**D**) 105, and (**E**) 120 min.

**Figure 3:**
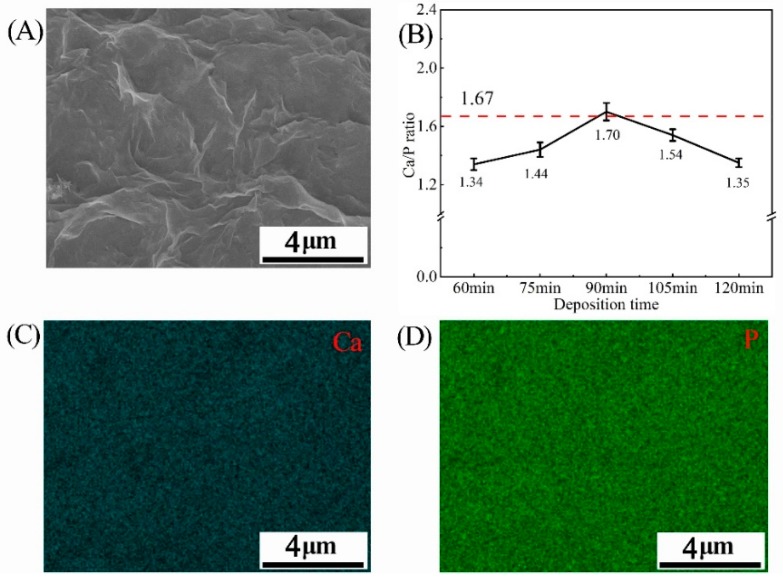
(**A**) SEM images of a dense and uniform surface of HA coating at deposition time of 90 min; (**B**) Ca/P ratio curve of HA coating on PVA/PLA braids at various deposition time; (**C**,**D**) EDS mapping analyses at 90 min.

The application of electrochemical deposition provides a dense and uniform HA coating on the rugged surface of PVA/PLA braids, as shown in Figure 2. The SEM observation of the braid surface before deposition is shown in Figure S1. At the deposition time of 60 min, the HA crystal was mainly in the form of micron-sized particles. The diameter of HA crystals can be observed in Figure S2, which is about 1.5 μm. This implies that this is the initial stage of electrodepositing the HA crystals [25]. Then, with the increase of time, the particles aggregate and extrude each other to form flat-like HA crystals with a smooth and compact plane. When excessively deposited, circular particles are grown on the HA flat. The EDS mappings at 90 min show that the distribution of calcium and phosphorus elements of the HA flat was uniform which just proves the uniformity of HA deposition (Figure 3A,C,D).

**Figure 4:**
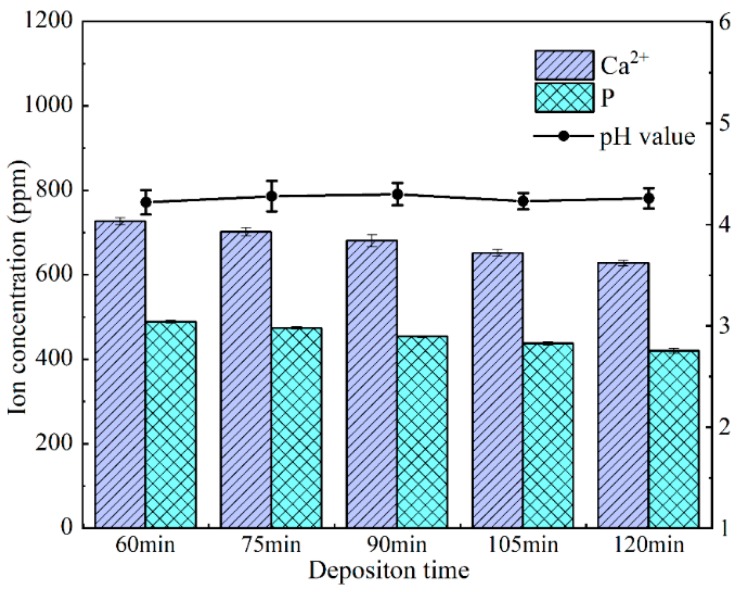
Calcium ion concentration, total phosphorus content and pH value of electrolyte as related to deposition time during electrodeposition.

The closer the calcium and phosphorus element ratio (Ca/P ratio) is to 1.67 (Ca/P ratio of pure HA), the less other calcium phosphate phases are contained in the HA coating, which can also reflect the crystallinity of HA. Table S1 in the supporting information is the Ca/P ratio of HA coatings obtained by electrodeposition in the past two years by other authors and the values are mostly between 1.55 and 1.70. In this study, the Ca/P ratio of the HA coating gradually increases to 1.34, 1.44, and 1.70 with the increase in deposition time. However, a deposition of 120 min adversely affects the Ca/ P ratio, which drops down to 1.30 (Figure 3B). The phenomenon may be attributed to calcium deficiency. Figure 4 shows the change in calcium ion concentration in the electrolyte by ICP-AES and reveals that with the increase of time, the calcium ions concentration declines near the cathode, which participate in Equation (5) to form hydroxyapatite. An excessive deposition time decreases the calcium ion concentration, which in turn results in a lower Ca/P ratio [26].

**Figure 5:**
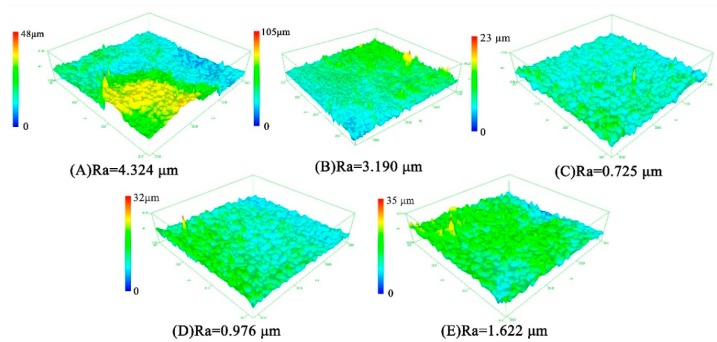
Average surface roughness (Ra) and 3D profilometry of HA coating on PVA/PLA braids with deposition time of (**A**) 60, (**B**) 75, (**C**) 90, (**D**) 105, and (**E**) 120 min.

The low roughness of the HA coating facilitates cell attachment [27,28]. The variation trend of average surface roughness of HA coatings is consistent with the SEM images. Roughness of HA coating after 90 min-deposition reached 0.725 μm, which is four times better than that after 60 min deposition. However, when the deposition time exceeds 90 min, the surface roughness increases slightly. Figure 5 reveals that the increase of deposition time could improve the average surface roughness (Ra).

### 3.2. The Chemical Bonds and Phase Composition of HA Coatings

**Figure 6:**
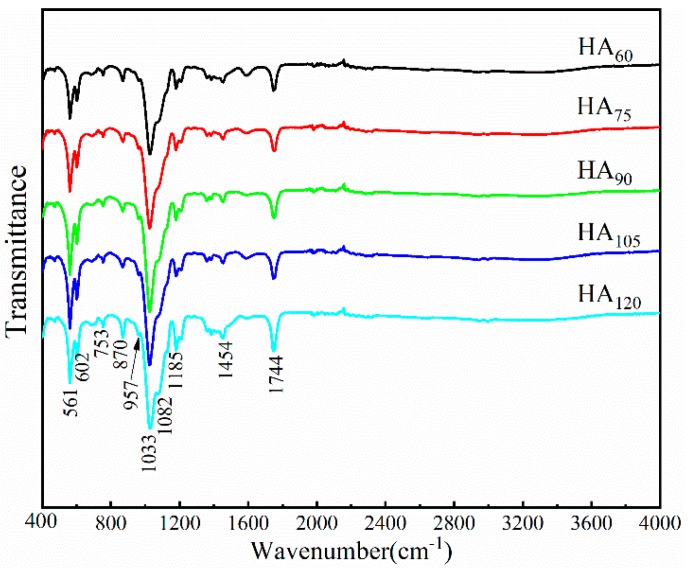
FTIR spectrum of HA coating on PVA/PLA braids at different deposition time.

Figure 6 reveals the typical FTIR characteristic peaks of HA on PVA/PLA braids, involving P-O bending vibrations (υ4) of PO_4_^3−^ at 602 cm^−1^ and 561 cm^−1^, the P-O stretching vibrations (υ3) of PO_4_^3−^ at 957 cm^−1^ and 1033 cm^−1^, the internal hydroxyl band at 3230~3550 cm^−1^. The PVA/PLA braids as a substrate also show some characteristic peaks, such as 1185 cm^−1^(carboxyl group in PLA), 1377, 1457 and 1745 cm^−1^ (methyl group in PLA/PVA). Meanwhile, 870 cm^−1^ (CO_3_^2−^) means the B-type or A-type carbonated hydroxyapatite (CHA), suggesting that a small amount of PO_4_^3−^ is replaced by CO_3_^2−^ produced by the carbon dioxide in the air dissolving in water [29]. In Table 1, we list some chemical bonds and phases corresponding to the characteristic peaks in Figure 6.

**Figure 7:**
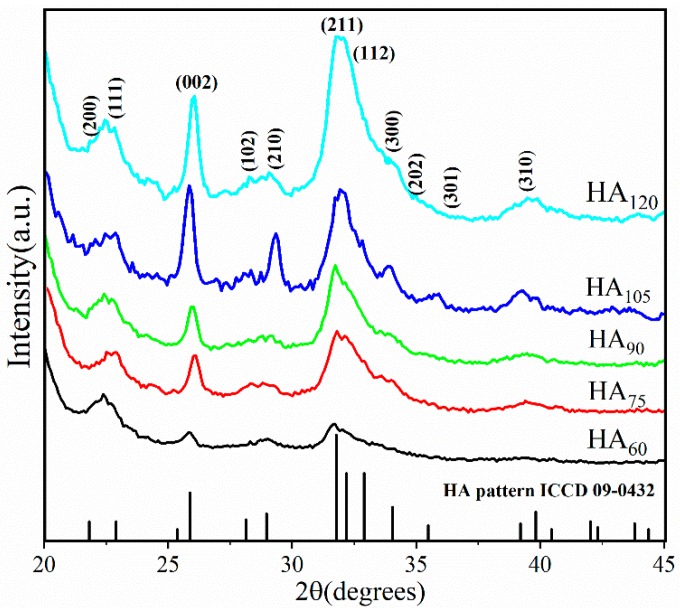
XRD patterns of HA coating on braid at different deposition time.

Figure 7 shows the XRD patterns of HA coatings on the PVA/PLA braid surface at various deposition times (60, 75, 90, 105 and 120 min). All the diffraction peaks are consistent with the ICDD database diffraction file #09-0432, which proves that the highly crystalline HA coating is obtained by electrodeposition on the PVA/PLA braid surface. We also compared the relevant standard files for the possible calcium phosphate, such as dicalcium phosphate dihydrate (DCPD, CaHPO_4_·2H_2_O, #09-0077), tri-calcium phosphate (TCP, Ca_3_(PO_4_)_2_, #09-0169) and Calcium carbonate (CaCO_3_, #17-0763). None of the main diffraction peak of substances mentioned above is found in the XRD patterns from the coatings obtained in this experiment, indicating HA coating presents a single-phase crystal via electrodeposition. The significant diffraction peaks at 2 theta degrees of 22.9°, 25.9°, 28.9°, 31.8° and 32.2° correspond to the HA crystallographic plane (111), (002), (210), (211) and (112), respectively.

The increase in diffraction peak intensity also implies that prolonging deposition time elevates the crystallinity of HA coating. The estimated crystallinity degree increases from 33.30 to 57.55% when deposition time increases from 60 min to 120 min. Meanwhile, the grain size of crystals obtained after a 90 min deposition is computed using the Debye–Scherrer equation according to Ref. [24] and shown in Table 2. The average grain size of HA coating is 10.10 nm, which is one half as small as the average grain size (20 nm) obtained by the precipitation method [24]. In particular, at a crystallographic plane (0 0 2), the grain size is 15.42 nm, which is distinctively larger than that of other crystallographic planes. This result suggests that the formation of HA crystals shows a preferred orientation along the *c*-axis [38]. Table 3 shows calculated cell parameters of HA crystals at different deposition time. Compared to the standard cell parameters of HA crystals according to the ICDD database diffraction file #09-0432, the value of the calculated c (Å) of HA crystals prepared by electrodeposition slightly increases, and a slight decrement of a (Å)/b (Å) is detected. Moreover, with the increase of deposition time from 60 min to 120 min, the unit cell parameter a (Å)/b (Å) decreases first and then increases, while the value of c (Å) increases first and then decreases [39].

### 3.3. The Formation Mechanism of HA Coatings Prepared by Electrodeposition

Formation mechanism of hydroxyapatite based on chemical reaction is showed as follows.

(1) $$2H_{2}O+2e^{-}\to H_{2}↑+2OH^{-1}$$

(2) $$H_{2}O_{2}+2e^{-}\to 2OH^{-}$$

(3) $$H_{2}PO_{4}^{-}+OH^{-}\to HPO_{4}^{2-}+H_{2}O$$

(4) $$HPO_{4}^{2-}+OH^{-}\to PO_{4}^{3-}+H_{2}O$$

(5) $$10Ca^{2+}+6PO_{4}^{3-}+2OH^{-}\to Ca_{10}{\left(PO_{4}\right)}_{6}{\left(OH\right)}_{2}$$

The presence of bubbles nearby the cathode is observed, which is consistent with the hydrogen production shown in Equation (1). According to Equations (1) and (2), hydroxyl radicals are generated. Following the increase in hydroxide ion concentration, both of the hydrogen phosphate ions and phosphate ions have a greater concentration (Equations (3) and (4)). Simultaneously, calcium ions move to the proximity of the cathode and start reacting with phosphate groups and hydroxyl radicals to generate HA (Equation (5)) [40]. The production of a small amount of carbonate (CO_3_^2−^) detected in the FTIR spectrum is as follows:

(6) $$CO_{2}+H_{2}O\to H_{2}CO_{3}$$

(7) $$H_{2}CO_{3}+OH^{-}\to HCO_{3}^{-}+H_{2}O$$

(8) $$HCO_{3}^{-}+OH^{-}\to CO_{3}^{2-}+H_{2}O$$

Phosphate in HA is easily replaced by carbonate (CO_3_^2−^) which is generated by carbon dioxide dissolved in water, forming carbonized hydroxyapatite (CHA) [41,42]. However, this process is so weak that the presence of CHA cannot be observed in XRD patterns. In fact, the large amount of hydroxyl radicals produced in Equations (1) and (2) can also promote the formation of phosphoric acid groups and HA in Equations (3)–(5).

**Figure 8:**
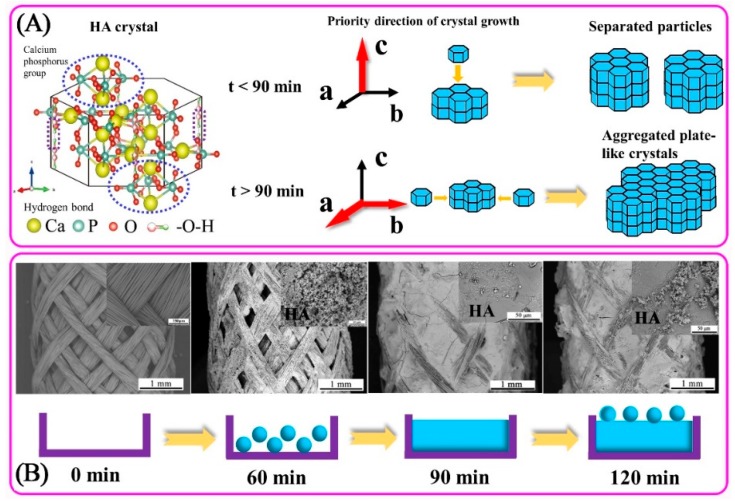
(**A**) The formation mechanism of granular and plate-like HA crystals during electrodeposition, in which the crystal structure of HA is drawn by Vesta 3.0 software and the red arrow is the preferred growth direction of HA crystal. (**B**) SEM images of braids at different deposition times and its simple models, in which the surface of braid can be simplified to a rectangle with a notch in the upper part before deposition (0 min).

Figure 8A shows the schematic diagram of the forming mechanism of granular and plate-like HA crystals during electrodeposition. The HA crystal is a regular hexagonal prism and belongs to the p63/m hexagonal space group. The ball-and-stick model diagram shows that the calcium ion and the phosphate group are located at upper and lower planes of hexagonal prism, and the hydroxyl group lies on the left and right sides of the prism portion. The hydrogen bond between the hydroxyl groups forms the connection to the HA unit cell, while the calcium phosphate group connects with the HA upper- and lower-unit cells [43,44].

In Figure 8B, the rugged PVA/PLA braid served as the substrate for electrodeposition is simplified into a rectangular model with an upper notch (0 min). Deposition time before 60 min belongs to the nucleation stage of HA crystals, and HA particles form very little. There is still a large part of space to be filled inside the model. After 60 min, it belongs to the growth period of HA crystal, and the HA crystal grows rapidly. When the deposition time increases to 90 min, all the indentation of the banding has been filled. The HA particles are formed on the HA coating surface again at 120 min, which is consistent with the change of HA surface morphology in SEM images (Figure 2).

At the initial stage of deposition (<60 min), HA crystals nucleate on the braid surface; after nuclei completely covers with the surface of PVA/PLA braid (>60 min), HA crystals begin to grow, and then aggregated to a smooth plate-like plane; when deposited for long time (>90 min), circular HA particles are formed on the plate-like plane again, resulting in a slight increase in roughness (Figure 5E) [45,46]. The hydroxyl radicals produced by electrolysis react with phosphate and calcium ions to form HA immediately. Such that no obvious fluctuation in the pH value is found during electrodeposition, indicating that the concentration of hydroxyl radicals in electrolyte is stable (Figure 4).Calcium ion and total phosphorus content in electrolyte as related to deposition time show that when deposition time is less than 90 min, there is high concentration of calcium and phosphorus ion, and the HA crystals thus preferentially grow along the c-axis. This outcome conforms well to the high intensity of diffraction peak of (002) crystal plane in XRD (Figure 7). However, when exceeding 90 min, calcium ion concentration and total phosphorus content in the electrolyte decreases, and the crystals grow preferentially along the a- or b-axis. At this time, a new diffraction peak is appeared on (211) (210) (301) crystal planes, thus forming the plate-like HA morphology [47].

## 4. Conclusions

In conclusion, HA coating is successfully electrodeposited on the rugged surface of a PVA/PLA braid. Deposition time affects the dynamic crystallization process of electrodeposited HA, and proper deposition time helps to improve the quality of HA coating. The main conclusions in this paper are as follows:

(a) With the increase of time, the particles aggregate and extrude each other to form flat-like HA crystals with a smooth and compact plane. The Ca/P ratio of the HA coating gradually increases to 1.34, 1.44, and 1.70 with the increase in deposition time. Roughness of HA coating after 90 min-deposition reaches 0.725 μm, which is 4 times better than that after 60 min deposition.
(b) Crystallinity degree increases from 33.30% to 57.55% when deposition time increases from 60 min to 120 min. Moreover, HA crystal shows a preferred orientation along the c-axis. Correspondingly, the unit cell parameter a (Å)/b (Å) decreases first and then increases with deposition time, while the value of c (Å) increases first and then decreases.
(c) The forming mechanism of HA coating shows as follow. At the initial stage of deposition (<60 min), HA crystals nucleate on the braid surface; after nuclei completely covers with the surface of PVA/PLA braid (>60 min), HA crystals begin to grow, and then aggregated to a smooth plate-like plane; When deposited for a long time (>90 min), circular HA particles are formed on the plate-like plane again, resulting in a slight increase in roughness.

Resultantly, 90 min-deposition generates optimal HA coating which is dense, uniform and highly crystallized, and has Ca/P ratio of 1.70, average roughness of 0.725 μm. This work will provide new ideas for the preparation of bioactive ceramic coatings on biodegradable materials for bone implants.

## Figures and Tables

**Table 1:** Assignment of FTIR spectra of PVA/PLA-HA braid presented in Figure 6.

Phase	IR Absorption Bands (cm^−1^)	Description	Ref.
P-O	561,602	bending vibrations (υ4)	[30]
	957,103.3	stretching vibrations (υ3)	
-CH_2_	753	PVA	[31]
CO_3_^2−^	870	carbon dioxide dissolving in water	[32]
CH_2_-OH	1082	PVA/PLA, stretching vibrations	[33,34]
C-OH	1185	PVA, stretching vibrations	[35]
C-H	1,385,145.4	deformation vibrations	[22]
C=O	1744	PLA, stretching vibrations	[36]
-OH...HO-	3230~3550	internal hydroxyl band	[37]

**Table 2:** Grain size and Miller indices of main 2-Theta angles of HA crystals obtained after a 90 min deposition.

2-Theta (deg)	Grain Size (nm)	Miller Indices (*h k l*)	Standard 2-Theta (deg)
22.862	9.62	(1 1 1)	22.752
25.863	15.42	(0 0 2)	25.298
31.778	7.37	(2 1 1)	31.839
39.198	7.97	(1 2 2)	39.253
Average	10.10 ± 5.32	—	—

**Table 3:** Calculated cell parameters of HA crystals obtained at different deposition time.

Sample	a (Å)	b (Å)	c (Å)
HA_60_	9.3902	9.3902	7.0644
HA_75_	9.3531	9.3531	7.0832
HA_90_	9.2818	9.2818	7.2429
HA_105_	9.3318	9.3318	7.1110
HA_120_	9.3799	9.3799	7.1220
Standard HA	9.4180	9.4180	6.8840

## Supplementary Materials

The following are available online at https://www.mdpi.com/2079-4991/9/5/679/s1, Table S1. The Ca/P ratio of HA coatings obtained by electrodeposition in the past two years by other authors; Figure S1. Surface morphology of undeposited braids, (A) SEM image of ×40 magnifications (B) SEM image of ×1000 magnifications; Figure S2. HA Crystal Diameter at 60 min Electrodeposition.
