# Supplementary material for: Properties and Mechanism of Hydroxyapatite Coating Prepared by Electrodeposition on a Braid for Biodegradable Bone Scaffolds
Source: Nanomaterials (Basel). 2019 May 2;9(5):679. doi: 10.3390/nano9050679 (PMC6567105; doi:10.3390/nano9050679)
Supplement: Supplementary file 1 [file nanomaterials-09-00679-s001.pdf]

nanomaterials

Supporting Information

Properties and Mechanism of Hydroxyapatite Coating Prepared by Electrodeposition on a Braid for Biodegradable Bone Scaffolds

Ting-Ting Li [1,2,3], Lei Ling [1], Mei-Chen Lin [4,5], Qian Jiang [1,2], Qi Lin [6], Jia-Horng Lin [1,3,4,5,7,8,9,*] and Ching-Wen Lou [3,7,8,10,11,*]

[1] Innovation Platform of Intelligent and Energy-Saving Textiles, School of Textile Science and Engineering, Tianjin Polytechnic University, Tianjin 300387, China; tingtingli@tjpu.edu.cn (T.-T.L.); 1731015027@stu.tjpu.edu.cn (L.L.); jiangqian@tjpu.edu.cn (Q.J.)
[2] Tianjin and Ministry of Education Key Laboratory for Advanced Textile Composite Materials, Tianjin Polytechnic University, Tianjin 300387, China
[3] Fujian Key Laboratory of Novel Functional Fibers and Materials, Minjiang University, Fuzhou 350108, China
[4] School of Chinese Medicine, China Medical University, Taichung 40402, Taiwan; ritalin2870@mail.fcu.edu.tw
[5] Laboratory of Fiber Application and Manufacturing, Department of Fiber and Composite Materials, Feng Chia University, Taichung 40724, Taiwan
[6] Fujian Engineering Research Center of New Chinese Lacquer Material, Minjiang University, Fuzhou 350108, China; linqi@mju.edu.cn
[7] Ocean College, Minjiang University, Fuzhou 350108, China
[8] College of Textile and Clothing, Qingdao University, Shandong 266071, China
[9] Department of Fashion Design, Asia University, Taichung 41354, Taiwan
[10] Department of Bioinformatics and Medical Engineering, Asia University, Taichung 41354, Taiwan
[11] Department of Medical Research, China Medical University Hospital, China Medical University, Taichung 40402, Taiwan
* Correspondence: jhlin@fcu.edu.tw (J.-H.L.); cwlou@asia.edu.tw (C.-W.L.); Tel.: +886-424-518-661 (J.-H.L.

Table S1. The Ca/P ratio of HA coatings obtained by electrodeposition in the past two years by other authors.

Substrate	Electrolyte	Current Density/Voltage	Temperature	Time	PH value	Ca/P Ratio*	Ref.
Ti disc	0.61 mM $NH_4H_2PO_4$ and 0.36 mM $Ca(NO_3)_2{\cdot}4H_2O$	potentiodynamic mode, from −1.4 V to 0 V, scanning rate 0.2 mV/s	50/75 °C	2 h	5	1.63	[S1]
Ti64 alloy	$Ca(NO_3)_2NH_4H_2PO_4$ Electrolyte resistivity was 18 MΩ·cm at 25 °C	potentiodynamic mode, from −1.4 V to 0 V, scanning rate 0.2 mV/s	75 ± 0.5 °C	2 h	5/6	1.51	[S2]
Ti	0.008 M $Ca(NO_3)_2{\cdot}4H_2O$, 0.005 M $NH_4H_2PO_4$, 1 M $NaNO_3$ and 6 mL/L H_2O_2	15 mA/cm^2	65 ± 2 °C	---	6	1.64	[S3]
Carbon fibers	3.80 × 10^{-4} M $NH_4H_2PO_4$, 6.35 × 10^{-4} M $Ca(NO_3)_2$ and 0.1mol/L $NaNO_3$	1.0, 3.0, 5.0, 7.0 and 9.0 mA	98.4 °C	60,120,180 mins	6	1.57	[S4]
Nitinol alloy	0.042 M $Ca(NO_3)_2$, 0.025 M $(NH_4)_2HPO_4$, 6 mL/L H_2O_2/0.008 M $Ca(NO_3)_2$, 0.0005 M $(NH_4)_2HPO_4$, 6 mL/L H_2O_2	1.5、3、5 mA/cm^2, 1.5、5、15 mA/cm^2	70 ± 1°C	25 min	6	1.70	[S5]
Titanium plate	0.0006 M $Ca(NO_3)_2$, 0.00036 M $(NH_4)_2HPO_4$, 0.1 M $NaNO_3$/ 0.025 M $Ca(NO_3)_2$, 0.015 M $(NH_4)_2HPO_4$ 0.1 M $NaNO_3$	1 mA/cm^2	100 °C	2 h	6	1.67	[S6]

* The Ca/P ratio shown in the table is the best value obtained in the article.

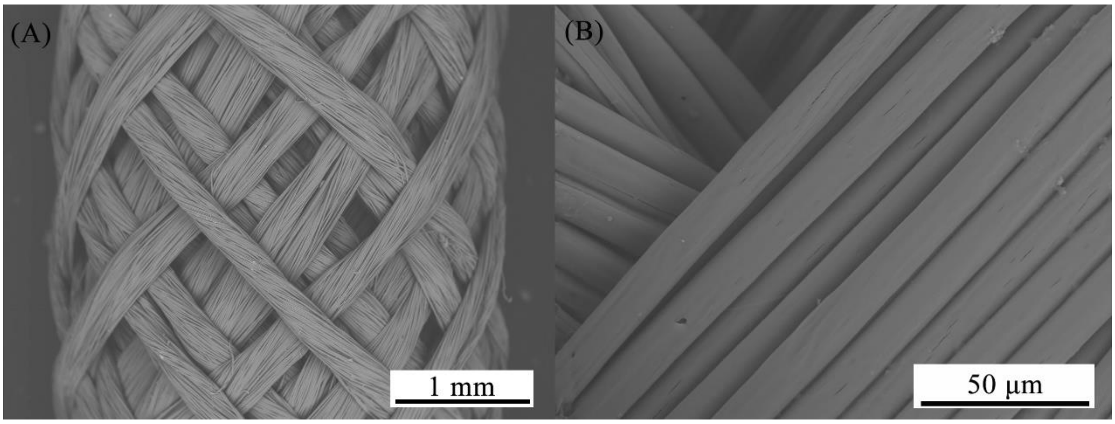

Figure S1. Surface morphology of undeposited braids, (**A**) SEM image of ×40 magnifications (**B**) SEM image of ×1000 magnifications.

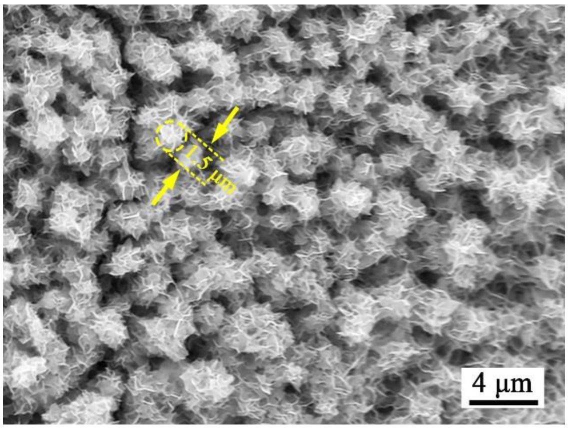

Figure S2. HA Crystal Diameter at 60 min Electrodeposition.

References

[S1] Cotrut, C.M.; Vladescu, A.; Dinu, M.; Vranceanu, D.M. Influence of deposition temperature on the properties of hydroxyapatite obtained by electrochemical assisted deposition. *Ceramics International* **2017**, *44*, 669–677

[S2] Vladescu, A.; Vranceanu, D.M.; Kulesza, S.; Ivanov, A.N.; Bramowicz, M.; Fedonnikov, A.S.; Braic, M.; Norkin, I.A.; Koptyug, A.; Kurtukova, M.O.; et al. Influence of the electrolyte's pH on the properties of electrochemically deposited hydroxyapatite coating on additively manufactured Ti64 alloy. *Scientific Reports* **2017**, *7*, 16819.

[S3] Fathyunes, L.; Khalil-Allafi, J. Effect of employing ultrasonic waves during pulse electrochemical deposition on the characteristics and biocompatibility of calcium phosphate coatings. *Ultrasonics Sonochemistry*. **2018**, *42*, 293–302.

[S4] Liu, Q.; Zhang, C.; Bao, Y.; Dai, G. Carbon fibers with a nano-hydroxyapatite coating as an excellent biofilm support for bioreactors. *Applied Surface Science* **2018**, *443*, 255–265.

[S5] Marashi-Najafi, F.; Khalil-Allafi, J.; Etminanfar, M.R. Biocompatibility of hydroxyapatite coatings deposited by pulse electrodeposition technique on the Nitinol superelastic alloy. *Materials Science and Engineering: C* **2017**, *76*, 278–286.

[S6] Pang, S.M.; He, Y.; He, P.; Luo, X.S; Guo, Z.Z.; Li, H. Fabrication of two distinct hydroxyapatite coatings and their effects on MC3T3-E1 cell behavior. *Colloids and Surfaces B-Biointerfaces* **2018**, *171* 40–48.
